# Long-term increases in wing length occur independently of changes in climate and climate-driven shifts in body size

**DOI:** 10.1098/rspb.2024.2556

**Published:** 2025-01-22

**Authors:** Tiffany Dias, Nathan P. Lemoine, Scott W. Yanco, Marketa Zimova, Rachael A. Bay, Brian C. Weeks

**Affiliations:** ^1^ School for Environment and Sustainability, University of Michigan, 440 Church Street, Ann Arbor, MI 48109, USA; ^2^ Department of Biological Sciences, Marquette University, 1428 West Clybourn Street, Milwaukee, WI 53233, USA; ^3^ Department of Biological Sciences, Ohio University, 57 Oxbow Trail, Athens OH 45701, USA; ^4^ Department of Evolution and Ecology, University of California, One Shields Avenue, Davis, CA 95616, USA

**Keywords:** allometry, birds, body size, climate change, morphology, wing length

## Abstract

Recent widespread reductions in body size across species have been linked to increasing temperatures; simultaneous increases in wing length relative to body size have been broadly observed but remain unexplained. Size and shape may change independently of one another, or these morphological shifts may be linked, with body size mediating or directly driving the degree to which shape changes. Using hierarchical Bayesian models and a morphological time series of 27 366 specimens from five North American migratory passerine bird species, we tested the roles that climate and body size have played in shifting wing length allometry over four decades. We found that colder temperatures and reduced precipitation during the first year of life were associated with increases in wing length relative to body size but did not explain long-term increases in wing length. We found no conclusive evidence that the slope of the relationship between body size and wing length changed among adult birds in response to any climatic variable or through time, suggesting that body size does not mediate shifts in relative wing length. Together, these findings suggest that long-term increases in wing length are not a compensatory adaptation mediated by size reductions, but rather are driven by non-climatic factors.

## 1. Background

While warming temperatures have been associated with reductions in body size across a diversity of species [1–3], the consequences of these changes remain unknown. As body size shrinks through time, shifts in other morphological traits are expected to occur because traits covary in consistent ways among individuals of the same developmental stage within species (i.e. static allometry) [4–6], and allometric relationships are generally thought to be strongly constrained [4,7,8]. However, allometry can evolve in response to natural selection [9–12] and contemporary shifts in environmental conditions may alter the selective landscape across space or time in ways that drive allometric evolution despite constraints within species [13,14]. For example, temperature has been found to affect the allometric relationship between mass and limb length in arthropods, with relatively longer limbs in warmer environments [15]. Thus, if body size mediates the strength of selection that is imposed by climate change on morphological traits, reductions in body size may precipitate adaptive changes in other traits that coincide with shifts in allometric relationships (i.e. shape). Given that shifts in shape have occurred contemporaneously with changes in size in many taxa [16–23], this raises the question: are these changes in shape simply occurring in parallel with, but independently of changes in size, or are they causally linked to warming-driven body size reductions?

Climate change may impose selection on shape directly in response to shifting thermoregulatory demands, energetic and water expenditures, and resource acquisition costs. As temperatures warm, the capacity to dissipate heat decreases [24], energy costs to maintain body temperature may rise [25,26] and water loss is greater [27–29]. Across a range of species, appendage length has increased as temperatures have warmed [30] and in birds, heat dissipation demand has been proposed to explain increased wing bone length in warmer climates [31]. Wing bone length is highly correlated with the length of the primary flight feathers in passerines [31], thus increasing flight feather length through time may be a secondary outcome of increases in wing bone length. Longer wings that result from increases in feather length may also be advantageous and directly selected for in warmer conditions as associated increases in flight efficiency [32] may compensate for exacerbated rates of water loss and energy expenditure. Changes in precipitation, which positively impacts resource availability [33–35], have also been linked to trends in body size [36,37] and shape [17,36–38]. In birds, reductions in precipitation have been associated with increases in wing length relative to body size (i.e. reduced wing loading), which allow birds to fly further and thus compensate for increased time and energy costs associated with obtaining resources in the context of resource scarcity [17]. If wings are under selection for thermoregulation or flight efficiency, warming temperatures or drier conditions may thus directly alter the relationship between body size and wing length such that birds of all sizes have relatively longer wings post-selection.

Alternatively, shifts in shape (e.g. longer wings), whether driven by climate or not, may be exacerbated or mitigated by other traits, such as body size, and thus require the relationship between traits to shift within species in response. Because body size is integral to species’ life histories [39–41], changes in body size that are occurring in response to warming are expected to have fitness consequences [42–45] that may require or obviate compensatory adaptations, such as shifts in other morphological traits. In endotherms, fundamental aspects of physiology change with body size, including higher mass-specific metabolic demands in smaller individuals [46]. This may make energetically demanding tasks more difficult as individuals get smaller, unless mitigated by, for example, sufficient increases in metabolic efficiency [47] or increases in wing length to increase flight efficiency [32]. Additionally, reductions in appendage length that would accompany body size reductions, should allometry be preserved, can hinder individuals’ abilities to perform various activities, such as migrate [48–50], or compete for resources [51–53]. Smaller individuals also have higher rates of water loss relative to larger individuals [54] that would be exacerbated by warming temperatures and further increase the need for compensatory adaptation via shifts in shape. Alternatively, reductions in body size may mitigate selection for shifts in shape such that greater shape shifts are observed among larger individuals. Larger individuals may require greater increases in heat dissipation capacity [55] as temperatures warm or may be particularly vulnerable to climate-driven resource shortages than smaller individuals because they have greater resource needs [56].

**Figure 1.**
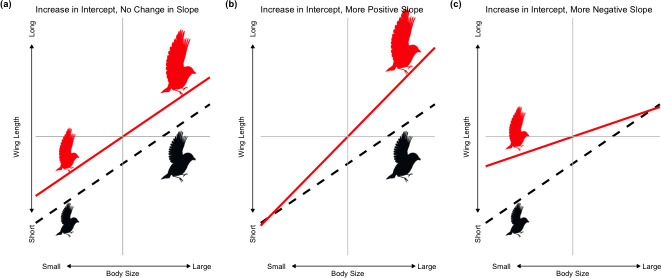
Possible shifts in allometry that coincide with longer wings. Allometric relationships between log-transformed traits can be defined as a line with a slope and intercept term. Here, the allometric intercept represents the mean wing length at the mean body size of the species, while the allometric slope is a measure of how wing length varies with body size. The black, dashed line represents the initial relationship between body size and wing length, while the red, solid line represents the shifted relationship between these two traits. (a)If the allometric intercept increases without a change in the slope, body size does not influence the degree to which wing length increases. (b)If the allometric intercept increases and the slope becomes more positive, body size plays a role in mediating increases in wing length such that larger individuals experience greater increases in wing length than smaller individuals. (c)If the allometric intercept increases and the slope becomes more negative, body size mediates increases in wing length such that smaller individuals experience greater increases in wing length than larger individuals.

Understanding how allometric relationships have shifted can help determine whether changes in shape (e.g. wing length allometry) have occurred independently of shifts in climate, have been driven by climate independently of size or are the outcomes of size-dependent selection imposed by climate (i.e. strength of climate-imposed selection on shape depends on body size; figure 1). North American migratory birds have become smaller as temperatures have risen over the past 40 years, but relative wing length has increased for these species [16,19], which suggests shifts in wing length allometry. Explanations for the contemporary increases in relative wing length have remained elusive: increases in wing length do not appear to be driven by advancing migratory phenology [57] and are unlikely to be explained by changes in migratory distance given that they have been observed both in migratory pathways [16,58] and in non-migratory Amazonian species [17]. Identifying the mechanistic basis of increases in relative wing length is an opportunity to develop a more holistic understanding of climate-driven morphological change and its limits.

Here, we take advantage of a densely sampled multi-species morphological time series dataset to investigate how different aspects of climate predict shifts in the relationship between wing length and body size and when changes in relative wing length occur during the annual cycle. We further test whether such shifts are dependent on or occur independently of changes in body size. To understand the direct effects of climate on relative wing length (figure 1*a*), we test three hypotheses: (A1) warmer temperatures drive increases in wing length to compensate for increased thermoregulatory demands; (A2) reductions in precipitation drive increases in wing length to compensate for reduced resource availability; and (A3) non-climatic factors drive long-term increases in wing length. To understand the role of size in mediating increases in relative wing length driven by climatic (hypotheses A1 and A2) or non-climatic (hypothesis A3) factors, we test three hypotheses: (B1) climate-driven selection for increased wing length acts more strongly on larger individuals to compensate for insufficient adaptation to climate change via body size reductions (figure 1*b*); (B2) climate-driven selection for increased wing length is stronger among smaller individuals to compensate for the non-thermoregulatory costs of climate-driven reductions in size (figure 1*c*); and (B3) non-climatic factors drive shifts in the slope of the relationship between wing length and body size through time (figure 1*b*,*c*). Understanding whether warming-associated size reductions are responsible for changes in shape will improve our understanding of how species adapt to climate change and what may constrain such adaptation.

## 2. Methods

### (a) Morphological data

Our morphological dataset includes 27 366 museum specimens collected from 1981 to 2016 [16], representing five species of North American migratory birds: dark-eyed junco (*Junco hyemalis*), swamp sparrow (*Melospiza georgiana*), song sparrow (*Melospiza melodia*), ovenbird (*Seiurus aurocapilla*) and white-throated sparrow (*Zonotrichia albicollis*). The specimens comprising our dataset were salvaged after fatal collisions with buildings in Chicago, IL, USA during their autumn or spring migrations. Individuals collected in autumn were either hatch-year (HY; i.e. individuals collected during their first migration) or after hatch-year (AHY; i.e. individuals collected after completing their first migration, in at least their second calendar year). All individuals collected in spring were considered AHY, as more specific ageing was not possible. All five species have declined in size and increased in wing length through time [16]. Before preparing each individual as a museum specimen, a single person measured the tarsus length and the relaxed wing length (the distance from the carpal joint to the tip of the longest primary flight feather on the folded wing) on each specimen [16]. Within this dataset, changes in tarsus are qualitatively similar to changes in mass and a multivariate index of size, but tarsus length is better able to capture subtle intra-specific changes in size through time [16], so we use tarsus length as an index of body size [59].

These five species were selected from a broader dataset of 52 North American migratory bird species [16] because they were the only species to have at least 3500 specimens with data on tarsus length, wing length, age and sex, thus ensuring sufficient and consistent sampling through time within each species for modelling annual changes in morphology. We filtered out any specimens with either tarsus or wing length measurements more than five median absolute deviations [60] from the median. To model allometry, tarsus and wing length measurements were first log-transformed. Then, these measurements were scaled to have a mean of 0 and s.d. of 1. Scaling was performed for each species separately to reduce the impact of size differences across species and enable interpretation of the allometric intercept as mean wing length at mean body size.

### (b) Climate data

We obtained temperature and precipitation data during key periods (pre-breeding, breeding, wintering and migration) across the 40 years of the dataset. We considered June and December as the breeding and wintering periods, respectively, based on knowledge of these species’ annual cycles. Peak migration periods were determined for each species by calculating the mean ordinal collection date during the spring and autumn, and treating dates falling within one s.d. in either direction as the migratory period. The pre-breeding period for a given species included the 30 days following the end of the peak spring migration period that was determined for that species. The breeding, wintering, migratory and resident ranges for these species were estimated using range maps from BirdLife International [61] and cropped, following [16], to remove unlikely breeding destinations given known migratory pathways and that the birds we collected were migrating through Chicago. We evaluated whether our results were robust to our cropping decision by systematically expanding the potential breeding localities and refitting all models (see the electronic supplementary material, figure S1 and tables S9-S11).

We calculated the mean daily temperature (i.e. the average of the maximum and minimum daily temperature) and the mean daily precipitation in the species’ breeding range during its pre-breeding period (i.e. pre-breeding conditions), mean June temperature anomaly and precipitation in the species’ breeding range (i.e. breeding season conditions), mean December temperature anomaly and precipitation in the species’ wintering range (i.e. wintering season conditions), and mean minimum daily temperature in the species’ migratory range (i.e. minimum temperature was used because the species migrate at night) during its peak migration periods for each year from 1980 to 2017. All climate data were scaled to have a mean of 0 and s.d. of 1 within each species. Monthly mean temperature data were from the National Aeronautics and Space Administration Goddard Institute for Space Studies surface temperature anomaly dataset that presents anomalies relative to a 1951−1980 base period [62], daily temperature data were from the Climate Prediction Center (CPC) Global Unified Temperature dataset, monthly precipitation data were from the Global Precipitation Climatology Project dataset [63], and daily precipitation data were from the CPC Global Unified Gauge-Based Analysis of Daily Precipitation dataset [64]. These datasets were provided by the National Oceanic and Atmospheric Administration Physical Sciences Laboratory, Boulder, Colorado, USA (https://psl.noaa.gov).

### (c) Identifying key climatic variables

To identify the key climatic variables to include in our main models of allometry, we constructed phylogenetic mixed models that model the effects of climate on individual-level morphologies without estimating within-year allometric slopes and intercepts for each species (for more detail, see electronic supplementary material, Preliminary models). We then included the climatic variables identified as significant (i.e. having 95% confidence intervals that did not overlap with 0) in our main models, which test their importance in driving allometry using a hierarchical Bayesian framework.

We constructed three individual-level phylogenetic mixed models of wing length to determine which climatic factors are associated with increases in relative wing length and to test whether there are any significant interactions with body size. The first model uses HY specimens, the second uses AHY specimens collected during spring migrations, and the final model uses AHY specimens collected during autumn migrations. In all three models, we control for year as a proxy for non-climatic factors that may have driven changes in wing length through time. For the HY-only model (*n* = 11 731), we include breeding season temperature, breeding season precipitation and temperature during the peak autumn migration period as climatic variables. We also include the temperature and precipitation on the wintering grounds of the prior year, the minimum temperature during the peak spring migration period prior to the breeding season, and the temperature and precipitation during the pre-breeding period in order to test for the potential effects of those climatic variables on HY birds mediated through effects on parental condition. For the spring (*n* = 11 481) and autumn (*n* = 3122) AHY models, we include the pre-breeding period temperature and precipitation, breeding season temperature and precipitation, autumn migration temperature, wintering season temperature and precipitation, and spring migration temperature most recently experienced to the season when specimens were collected. In the spring AHY model, we also include the effects of temperature and precipitation during the wintering season prior to the most recent wintering season and the effect of temperature during the spring migration prior to the most recent spring season to test for sustained or potentially lagged effects. We similarly extend the autumn AHY model to include the wintering season conditions, spring migration conditions, pre-breeding period conditions, breeding season conditions and autumn migration conditions prior to the most recent seasons experienced by autumn AHY birds.

### (d) Testing whether climate drives changes in wing length allometry

In our three main models, allometric relationships between wing length and body size were modelled for a given class of individuals (HY, spring AHY or autumn AHY) using a hierarchical Bayesian framework that was implemented in Stan [65] using the *stan_model()* function in the ‘rstan’ package [66] in R [67]. By modelling allometry independently for each age class and season, we evaluate changes in the allometric relationship between wing length and body size, how this relationship changes over the annual cycle, and the importance of each climatic variable at each stage.

In each model, species-specific allometric intercepts and allometric slopes of the relationship between body size and wing length were simultaneously (i) estimated for each year of data, and (ii) modelled as a function of the climate variables found to be important in the individual-based models described above and year. By modelling the allometric intercept, we test whether increases in wing length (figure 1*a*) have been driven by warming temperatures (hypothesis A1), by reductions in precipitation (hypothesis A2) or by non-climatic factors (using year as a proxy; hypothesis A3). By modelling the allometric slope, we test whether such changes are mediated by body size. A positive association between the allometric slope and climatic drivers of wing length increases (figure 1*b*) would be consistent with hypothesis B1, a negative relationship (figure 1*c*) would be consistent with hypothesis B2, and an association between the slope and year would be consistent with hypothesis B3.

In each hierarchical Bayesian model, for each species-year combination, *y*, in our data, we estimate the parameters $\beta_{0}$ (allometric intercept) and $\beta_{1}$ (allometric slope) that define the relationship between body size and wing length, while controlling for sex, *s*, and the possibility that allometry varies with sex. We use individual log-transformed and scaled body size (*z*) and wing length (*w*) measurements for either HY or AHY birds collected in a given season:


(2.1) $$\hat{w}=\beta_{0,y}+\beta_{1,y}*z+n_{0,p}*s+n_{1,p}*s*z,$$

where $n_{0,p}$ and $n_{1,p}$ are the estimated effect of sex on the allometric intercept and on the allometric slope, respectively, for species, *p*. Priors were specified as:


(2.2) $$w\;∼normal\;\left(\hat{w},half-Cauchy\;(0,5)\right),$$

(2.3) $$\beta_{*}\;∼normal\;\left({\hat{\beta }}_{*}{,}_{y},half-Cauchy\;(0,5)\right),$$

(2.4) $$n_{*}∼normal\;\left(normal\;(0,2),\;half-Cauchy\;(0,5)\right).$$

We estimate the parameters *I*_0_ and *I*_1_ (both matrices of estimated effects of climate variables or year, *c*, for each species, *p*) that define how climate and year, $G_{y,c}$, impact the allometric intercept (i.e. parameter ${\hat{\beta }}_{0}$ estimated for each species-year combination) and the allometric slope (i.e. estimated variable ${\hat{\beta }}_{1}$ estimated for each species-year combination) respectively:


(2.5) $${\hat{\beta }}_{*,y}\;=I_{*,p,c}*G_{y,c},$$

where priors for each species-specific estimate of a variable, *i*, in *I*_0_ or *I*_1_ were specified as:


(2.6) $$I_{*,p,c=i}∼normal\;\left(\mu_{i},\sigma_{i}\right),$$

and the priors for all global estimates of climate effects and year, μ, and their s.d., σ, were specified as:


(2.7) μ∼normal(0,2),

(2.8) σ∼half−Cauchy(0,5).

HY allometry was estimated for 177 unique species-year combinations using 11 798 individuals, AHY spring allometry was estimated for 174 unique species-year combinations using 12 318 individuals, and AHY autumn allometry was estimated for 171 unique species-year combinations using 3250 individuals. These hierarchical Bayesian models did not control for phylogenetic relatedness because there was low phylogenetic signal in our preliminary phylogenetic mixed models (electronic supplementary material, table S7). Additionally, our phylogenetic mixed models confirmed that our results were not influenced by correlations among the predictor variables (electronic supplementary material, tables S4–S6). All hierarchical Bayesian models were run with four chains, with each chain run for 4000 iterations with the first 2000 iterations discarded as burn-in. Model convergence was confirmed with the Rhat statistic [68] and by examining parameter trace plots and posterior distributions.

## 3. Results

### (a) Spring migration, pre-breeding and wintering season temperatures do not have long-term impacts on wing length allometry

We do not include temperature during spring migration as a parameter in any of our main models of allometry because it does not have a significant effect in any of the phylogenetic mixed models (electronic supplementary material, tables S4–S6). In our main model of HY allometry (table 1), we find that the wintering season temperature prior to the autumn when HY birds were collected is significantly and positively associated with the allometric slope (*β* = 0.02, probability of direction (pd) = 94.8%) but is not significantly associated with the allometric intercept (*β* = 0.01, pd = 76.1%). We also find that the pre-breeding temperature prior to when HY birds are collected has a significant and positive association with the allometric intercept (*β* = 0.05, pd = 98.7%), but no significant association with the allometric slope (*β* = −0.01, pd = 89.5%). However, temperatures during these periods do not have any significant associations with AHY allometry (electronic supplementary material, tables S5–S6).

**Table 1.** Effects of climate on HY wing length allometry are globally estimated from species-specific effects of each of our five species (bold indicates that the effect’s probability of direction is at least 90%). Species-specific effects in electronic supplementary material, table S1.

	term	mean	s.d.	probability of direction
allometric intercept	(intercept)	0.47	0.17	99.8% (+)
	prior wintering temperature	0.01	0.03	76.1% (+)
	**prior wintering precipitation**	**−0.03**	**0.02**	**94.1% (−**)
	**pre-breeding temperature**	**0.05**	**0.02**	**98.7% (+**)
	**pre-breeding precipitation**	**−0.02**	**0.02**	**91.6% (−**)
	**most recent breeding temperature**	**−0.06**	**0.02**	**99.1% (−**)
	**most recent breeding precipitation**	**−0.03**	**0.02**	**95.4% (−**)
	**year**	**0.18**	**0.02**	**100% (+**)
allometric slope	(intercept)	0.11	0.04	99.8% (+)
	**prior wintering temperature**	**0.02**	**0.02**	**94.8% (+**)
	prior wintering precipitation	0.01	0.04	66.9% (+)
	pre-breeding temperature	−0.01	0.01	89.5% (−)
	pre-breeding precipitation	0.00	0.02	61.7% (+)
	most recent breeding temperature	0.01	0.02	78.1% (+)
	most recent breeding precipitation	0.00	0.01	67.0% (+)
	**year**	**−0.02**	**0.01**	**92.5% (−**)

### (b) Colder breeding season temperatures during development are associated with increases in relative wing length

In our model of HY allometry (table 1), we find that breeding season temperature has a significant negative association with the allometric intercept of the relationship between body size and wing length (*β* = −0.06, pd = 99.1%) and no relationship with the allometric slope (*β* = 0.01, pd = 78.1%). The association with the allometric intercept is not significant among AHY birds collected during the following spring (allometric intercept *β* = −0.01, pd = 72.7%; allometric slope *β* = −0.01, pd = 83.5%; table 2), but it is significant again among AHY birds collected in the following autumn (allometric intercept *β* = −0.09, pd = 99.5%; allometric slope *β* = −0.01, pd = 75.1%; table 3). However, temperature during the breeding season most recent to when autumn AHY birds were collected is not significantly associated with wing length among autumn AHY birds (electronic supplementary material, table S6).

**Table 2.** Effects of climate on spring AHY wing length allometry are globally estimated from species-specific effects of each of our five species (bold indicates that the effect’s probability of direction is at least 90%). Species-specific effects in electronic supplementary material, table S2.

	term	mean	s.d.	probability of direction
allometric intercept	(intercept)	0.65	0.11	100% (+)
	**prior wintering precipitation**	**−0.02**	**0.02**	**92.7% (−**)
	**pre-breeding precipitation**	**−0.03**	**0.02**	**93.5% (−**)
	most recent breeding temperature	−0.01	0.04	72.7% (−)
	**most recent breeding precipitation**	**−0.03**	**0.03**	**90.1% (−**)
	**most recent autumn temperature**	**−0.04**	**0.03**	**94.8% (−**)
	most recent wintering precipitation	−0.01	0.02	79.7% (−)
	**year**	**0.19**	**0.04**	**99.9% (+**)
allometric slope	(intercept)	0.14	0.06	99.7% (+)
	prior wintering precipitation	0.01	0.03	71.1% (+)
	pre-breeding precipitation	−0.02	0.03	83.5% (−)
	most recent breeding temperature	−0.01	0.01	83.5% (−)
	most recent breeding precipitation	−0.01	0.03	71.2% (−)
	most recent autumn temperature	0.00	0.02	55.6% (+)
	most recent wintering precipitation	0.00	0.02	51.2% (+)
	year	−0.01	0.04	63.6% (−)

**Table 3.** Effects of climate on autumn AHY wing length allometry are globally estimated from species-specific effects of each of our five species (bold indicates that the effect’s probability of direction is at least 90%). Species-specific effects in electronic supplementary material, table S3.

	term	mean	s.d.	probability of direction
allometric intercept	(intercept)	1.00	0.14	100% (+)
	**prior wintering precipitation**	**0.03**	**0.03**	**90.0% (+**)
	**prior breeding temperature**	**−0.09**	**0.03**	**99.5% (−**)
	**prior breeding precipitation**	**−0.05**	**0.05**	**93.4% (−**)
	prior autumn temperature	−0.02	0.07	78.0% (−)
	most recent autumn temperature	0.03	0.04	88.6% (+)
	**year**	**0.19**	**0.07**	**99.8% (+**)
allometric slope	(intercept)	0.11	0.03	99.8% (+)
	prior wintering precipitation	0.02	0.03	88.7% (+)
	prior breeding temperature	−0.01	0.03	75.1% (−)
	prior breeding precipitation	−0.01	0.07	65.3% (−)
	prior autumn temperature	0.01	0.05	70.9% (+)
	**most recent autumn temperature**	**−0.03**	**0.05**	**90.3% (−**)
	year	0.02	0.03	75.9% (+)

### (c) Autumn migration temperatures have mixed effects on wing length allometry of after hatch-year birds

Temperature during autumn migration does not have a significant association with HY wing length (electronic supplementary material, table S4). In our model of spring AHY allometry (table 2), temperature during the autumn migration prior to when spring AHY birds were collected is significantly and negatively associated with the allometric intercept (*β* = −0.04, pd = 94.8%) and has no significant relationship with the allometric slope (*β* = 0.00, pd = 55.6%). Among AHY birds collected in the autumn of the next year (table 3), the association with the allometric intercept is no longer significant (allometric intercept *β* = −0.02, pd = 78.0%; allometric slope *β* = 0.01, pd = 70.9%). Additionally, temperature during the following autumn migration (i.e. the same period in which autumn AHY birds were collected) has no significant relationship with the allometric intercept of autumn AHY birds (*β* = 0.03, pd = 88.6%) but has a negative relationship with the allometric slope (*β* = −0.03, pd = 90.3%).

### (d) Drier wintering seasons and pre-breeding periods prior to development are associated with increases in relative wing length among hatch-year and spring after-hatch-year birds

In our HY allometry model (table 1), we find that precipitation during the wintering and pre-breeding periods prior to when HY birds hatch, develop and are collected are significantly and negatively associated with the allometric intercept (wintering precipitation *β* = −0.03, pd = 94.1%; pre-breeding precipitation *β* = −0.02, pd = 91.6%) but not associated with the allometric slope (wintering precipitation *β* = 0.01, pd = 66.9%; pre-breeding precipitation *β* = 0.00, pd = 61.7%). These associations are maintained among AHY birds collected in the following spring (table 2; allometric intercept (wintering precipitation) *β* = −0.02, pd = 92.7%; allometric intercept (pre-breeding precipitation) *β* = −0.03, pd = 93.5%; allometric slope (wintering precipitation) *β* = 0.01, pd = 71.1%; allometric slope (pre-breeding precipitation) *β* = −0.02, pd = 83.5%).

However, these associations are not maintained among AHY birds collected in the autumn over a year later (table 3). Precipitation during the wintering season prior to development has a positive association with the allometric intercept (*β* = 0.03, pd = 90.0%), while the relationship with the allometric slope is still not significant (*β* = 0.02, pd = 88.7%), and precipitation during the pre-breeding period prior to development is not associated with autumn AHY wing length (electronic supplementary material, table S6). Similarly, among spring AHY birds, precipitation during the most recent wintering season, which occurs after HY birds were collected, does not have a significant relationship with the allometric intercept (*β* = −0.01, pd = 79.7%) or allometric slope (*β* = 0.00, pd = 51.2%). Precipitation during the most recent wintering and pre-breeding periods are not associated with autumn AHY wing length (electronic supplementary material, table S6).

### (e) Drier breeding season conditions during development are associated with increases in relative wing length

In all our models of allometry, we consistently find a significant and negative association between precipitation during the breeding season prior to development and the allometric intercept (HY model: *β* = −0.03, pd = 95.4%; table 1; spring AHY model: *β* = −0.03, pd = 90.1%; table 2; autumn AHY model: *β* = −0.05, pd = 93.4%; table 3). The relationship with the allometric slope is not significant across all models (HY model: *β* = 0.00, pd = 67.0%; spring AHY model: *β* = −0.01, pd = 71.2%; autumn AHY model: *β* = −0.01, pd = 65.3%). However, in the autumn AHY phylogenetic mixed model, precipitation during the most recently experienced breeding season is not significant (electronic supplementary material, table S6).

### (f) Relative wing length increases through time but shifts in the allometric slope of the relationship between body size and wing length are not observed among after-hatch-year birds

In all our models of allometry, we consistently find a positive relationship between year and the allometric intercept (HY model: *β* = 0.18, pd = 100.0%; table 1; spring AHY model: *β* = 0.19, pd = 99.9%; table 2; autumn AHY model: *β* = 0.19, pd = 99.8%; table 3). Among HY birds, the positive association between year and the allometric intercept is accompanied by a negative association between year and the allometric slope (*β* = −0.02, pd = 92.5%). However, among AHY birds, the association between year and the allometric slope is not significant (spring AHY model: *β* = −0.01, pd = 63.6%; autumn AHY model: *β* = 0.02, pd = 75.9%).

## 4. Discussion

Changes in body size and body shape are consistent and widespread responses to global warming [1,2,30]. However, while evidence that warming temperatures can directly result in reductions in body size grows [3,69–74], the drivers of changes in body shape remain less well understood. We set out to test whether warmer temperatures drive increases in wing length to accommodate increased thermoregulatory demands (hypothesis A1), whether reduced resource availability resulting from reductions in precipitation could explain increases in wing length (hypothesis A2), whether climate-driven increases in wing length are mitigated by (hypothesis B1) or necessary to compensate for (hypothesis B2) warming-driven size reductions, and whether non-climatic factors play a role in driving long-term increases in wing length (hypothesis A3) or shifting the relationship between body size and wing length through time (hypothesis B3).

### (a) Lack of a consistent association between size-independent increases in wing length and warmer temperatures suggests increases in wing length are not adaptations to increased thermoregulatory demands

We find that warmer temperatures during the breeding season of development are associated with size-independent reductions in wing length (i.e. a more negative allometric intercept with no change in slope) that persist beyond birds’ first year of life. We also find that, among birds that completed an autumn migration, shorter wings are more likely to be observed, regardless of body size, when that autumn migration was warmer. These findings are inconsistent with the hypotheses that warmer temperatures drive increases in wing length (hypothesis A1) and that body size plays a role in mediating the relationship between temperature and wing length (hypotheses B1 and B2). Furthermore, our findings suggest that the breeding season of development and autumn migration are critical periods when temperature may drive size-independent shifts in wing length such that birds’ wings are relatively shorter when temperatures are warmer and relatively longer when temperatures are colder.

These results are unexpected given positive correlations between temporal increases in temperature and elongation of appendages that are found across taxa [30]. In birds and mammals, such associations have been proposed to be owing to developmental plasticity (reviewed in [75]). Alternatively, longer wings may be adaptive and selected for in warmer environments if associated increases in flight efficiency mitigate warming-driven increases in energy and water costs [28,32,55]. Longer appendages may also increase heat dissipation capacity [30,31,76]; the length of the relaxed wing chord (i.e. our wing length data) does not directly measure the length of the heat dissipating area of the wing, but it is tightly correlated with the length of the radius and ulna in passerines [31], such that lengthening of the wing chord may accompany increases in wing bone length. Though our finding that warmer pre-breeding temperatures are associated with size-independent increases in wing length among HY birds is consistent with such factors driving increases in wing length among our species (hypothesis A1), the fact that HY birds do not experience these temperatures themselves and the disappearance of this association among AHY birds together raise questions as to how pre-breeding temperatures could drive observed variation in HY wing length. Furthermore, the consistent negative relationship between breeding season temperature and wing length that we recover suggests that longer wings are not the result of adaptive changes to wing structure in response to warming temperatures.

Colder temperatures could result in increased wing length through developmental effects. Nestlings that develop in colder conditions may have lower body condition, smaller size, and lower energy reserves resulting from altered developmental dynamics [77–79], physiology [80–82] or biotic pressures [83,84], which could then lead to selection for increased flight efficiency via longer wings. In addition to effects driven by breeding season temperatures, colder temperatures during autumn migration may increase water loss as colder air is drier. Migrating birds actively alter flight altitude to avoid colder air [85] and retain water [86,87]. Thus, when colder temperatures require birds to fly at lower altitudes, where air resistance is higher [88], to conserve water, increases in flight efficiency may be needed to overcome the associated energetic costs.

### (b) Association between size-independent increases in wing length and drier conditions suggests increases in wing length are adaptive in the context of reduced resource availability

We find that reduced precipitation during the wintering and pre-breeding periods prior to development is associated with size-independent increases in wing length (i.e. a more positive allometric intercept with no change in slope) during birds’ first year of life. Furthermore, we find that longer wings are consistently associated with lower levels of breeding season precipitation, regardless of body size. These findings suggest that the wintering and pre-breeding periods prior to development and the breeding season of development are key periods when drier conditions may drive size-independent increases in wing length (hypothesis A2) but are inconsistent with a role for body size in mediating these shifts (hypotheses B1 and B2).

These findings are consistent with the hypothesis that reductions in precipitation (and associated resource limitation) select for compensatory increases in flight efficiency to reduce water loss or lower energy expenditure [17]. Specifically, our results suggest that reduced precipitation on the breeding grounds during development directly lead to longer relative wing length among HY birds, while reduced precipitation during the wintering season impacts HY birds during the following breeding season, potentially through impacts on parent condition. Rainfall and its impact on resource availability on the wintering grounds has been linked to body condition and departure for the breeding grounds among birds wintering in the tropics [89–91]; for temperate species, potential links between reduced precipitation and lower resource availability, such as reductions in snow cover driving declines in tree species [92], may have similar effects. Poor body condition of breeding birds owing to subpar wintering conditions may lead to lower provisioning rates of nestlings [93,94], which may then negatively impact nestling growth and condition [94,95]. Delayed departure for the breeding grounds and a shorter breeding season due to reduced rainfall may also result in a mismatch between peak demand for resources to provision nestlings and peak availability of resources [96,97], thus exacerbating this effect. The subsequent poor body condition and lower energy reserves of nestlings may then drive the increases in wing length observed among HY birds to generate adequate flight efficiency to survive.

### (c) Size-dependent effects of climate on wing length do not explain long-term shifts in wing length allometry

Two findings suggest that climate-associated increases in wing length are mediated by body size: warmer winter temperatures are associated with a more positive allometric slope among HY birds collected during the subsequent autumn migration and warmer temperatures during autumn migration are associated with a more negative allometric slope among AHY birds collected in that season. While potentially consistent with a role for body size mediating warming-driven increases in wing length (hypothesis B1 or B2), these changes in slope are not accompanied by a significant shift in the allometric intercept, and thus it is unclear what mechanism could be responsible for driving such changes. Because the wintering temperature effect is not observable by the following spring (i.e. in the spring AHY model), it also cannot explain long-term changes in wing length allometry, and the effect of autumn migration temperature observed among autumn AHY birds stands in contradiction to the associations found among AHY birds collected in the following spring or in the autumn of the next year. A potential explanation is that birds collected in autumn have not completed that autumn migration, so the total impact of autumn temperature on allometry may not be apparent. Indeed, the associations between temperature during completed autumn migration periods and allometry align, regardless of whether birds were collected in the autumn or spring. Therefore, the association between changes in slope among autumn AHY birds and the most recent autumn migration temperature is inconclusive.

### (d) Long-term increases in wing length are not driven by climate or by changes in body size

Taken together, our results suggest that warmer wintering, pre-breeding and autumn migration temperatures; colder breeding and autumn migration temperatures; and reductions in precipitation—while associated with changes in wing length within the annual cycle for subsets of the populations we assessed—do not appear to drive long-term increases in wing length in our species. The effects of pre-breeding temperature, wintering temperature, and wintering precipitation do not persist across age classes. We are reluctant to interpret the effect of the most recent autumn migration temperature on the allometry of autumn AHY birds as meaningful, given contrasting effects of temperature during previous, completed autumn migrations. While colder breeding and autumn migration temperatures are associated with longer wings, the direction of the temperature-wing length relationships and the long-term trends in temperature are inconsistent with a direct role for increasing temperatures in driving multi-decadal increases in wing length (electronic supplementary material, table S8). This is similarly true for reductions in breeding season precipitation. Additionally, climate only explains a small amount of the variation in wing length observed across age classes and through time. Finally, in all models, wing length increases through time regardless of body size and without simultaneous shifts in the allometric slope (inconsistent with hypothesis B3), even after accounting for the effects of climate. This suggests that non-climatic factors not included in our model are driving long-term changes in shape (consistent with hypothesis A3).

It is highly likely that selective pressures during migration shape wing length allometry to some degree, as mortality during migration is relatively high compared to other parts of the annual cycle [98] and all five species in our study are long-distance migrants. Migratory species tend to have longer and more pointed wings than non-migratory species [99,100] and longer wings are associated with better survival during migration [101] and longer migration distances [49,102]. Increases in flight efficiency may be helpful in reducing energy expenditure during migratory flight [103,104] and improving reproductive success on the breeding grounds as birds arrive earlier and in better condition [105,106]. If, through time, conditions during migration have become less favourable (e.g. through increasing storm frequency), larger ecological barriers have arisen as land use has changed and expanded, or stopover sites have been degraded, increased flight efficiency may be necessary for survival.

However, since long-distance migrants have also experienced contemporary reductions in wing length [101,107] and increases in wing length have been found alongside reductions in body size in non-migratory species as well [17], factors beyond migration must be important in driving shifts in shape. It is likely that such factors drive increases in wing length independently of body size, given that we found that the allometric slope of the relationship between body size and wing length does not change through time among AHY birds. One possible driver of size-independent increases in wing length is habitat fragmentation. Boreal forest songbirds have acquired longer wings in response to clear-cutting, while afforestation has been associated with wings becoming less pointed among temperate forest species [108], potentially in response to increased selection for longer wings as habitat quality and resource availability decline. Alternatively, reductions in predation pressure as populations of predators decline may relax selection for manoeuvrability [109], allowing for wings to become longer. However, while our dataset provides clear evidence of long-term increases in relative wing length, the species in our study, which are all long-distance migratory passerines, only represent a fraction of the diversity and variation present among North American migratory birds. Furthermore, we do not have knowledge of which populations our specimens belong to or what specific conditions they experience, which makes it difficult to determine what is driving observed morphological trends in our species. Identifying the non-climatic drivers of wing length increases remains critical for understanding the potential impacts and limits of this pronounced morphological trend and experimental work will be necessary to do so.

## 5. Conclusion

We find that climate influences shape during the first year of life for five species of long-distance migratory passerine birds, but these effects cannot explain long-term increases in wing length in our system. Similarly, we find no evidence to suggest that wing length increases are a compensatory response to warming-driven size reductions. Understanding the drivers and limitations underlying contemporary morphological adaptation and constraints on changes in allometry is key to determining potential limits to adaptation and species persistence.

## Data Availability

Code for reproducing our results is published at Zenodo [111] and is accessible via Dryad alongside data for all analyses, tables, and figures [110]. Supplementary material is available online [112].
